# Mini titanium plates; hearkening the end of non-rigid cranial bone flap fixation

**DOI:** 10.12669/pjms.334.12003

**Published:** 2017

**Authors:** Muhammad Junaid, Syed Sarmad Bukhari, Mobasher Ahmad Saeed, Mamoon Ur Rashid

**Affiliations:** 1 Lt. Col. Dr. Muhammad Junaid, FCPS. Associate Professor of Neurosurgery, Bahria University Medical and Dental College, Karachi, Pakistan; 2 Dr. Syed Sarmad Bukhari, MBBS. Khyber Teaching Hospital, Peshawar, Pakistan; 3 Surg. Cdre. Mobasher Ahmad Saeed, FCPS. Associate Professor of Anesthesiology, Bahria University Medical and Dental College, Karachi, Pakistan; 4 Dr. Mamoon Ur Rashid, MBBS. Department of Internal MedicineOrlando Hospital, FL, USA

**Keywords:** Craniotomy, Surgical Fixation Devices, Titanium, Surgical Procedure, Reconstructive

## Abstract

**Background and Objective:**

Craniotomy bone flaps should be replaced for both cosmetic and protective purposes. Different methods are available commercially. The aim of this study was to assess outcome of bone flap fixation using mini titanium plates and screws.

**Methods:**

Between March, 2011 and March, 2014, 71 patients underwent cranial bone flap fixation with mini titanium plates and screws after craniotomy and excision of supratentorial lesions at Combined Military Hospital, Peshawar. There were 42 males and 30 females with a mean age of 40.07. All patients had supratentorial lesions. Intracranial lesion size ranged from 3 cm by 2 cm to 7 cm by 5 cm. The changes of local incision and general condition were observed.

**Results:**

Subcutaneous effusion occurred in two patients. One patient developed a mild postoperative wound infection. CT scan showed good repositioning of the flap and edge to edge apposition at two weeks after operation. All the patients were followed up for 12 months post operatively. Skull had good appearance without any discharge and, local deformity or effusion. Repeat CT/MRI showed no subsidence or displacement of cranial flap or artifacts.

**Conclusion:**

Mini titanium plate and screw fixation of cranial flaps is a simple, cost effective and safe option for repositioning and immediate stability as compared to traditional sutures.

## INTRODUCTION

The biocompatibility and Osseo-integrative qualities of the element titanium have led to its widespread use in medicine as an in vivo implant. It has effectively replaced steel as the most commonly used metal in orthopedics and neurosurgery. Non-absorbable sutures were used to anchor the bone flap back into place following a craniotomy but they were associated with unwanted outcomes including functional and cosmetic defects as well as non-union and failure postoperatively.^1^ The craniofacial region is the most important part of the body with regards to harmony and symmetry and associated with self-image of the patient and modern day craniofacial surgery includes in itself the standard practice of maintaining this harmony and restoring the normal appearance for every patient to every possible degree. Every surgery is now planned with reconstruction in mind. Unfortunately, this has not been a priority amongst neurosurgeons, possibly because functional outcome has historically taken precedence over esthetic outcome.^2^ Besides the physical appearance, non-rigid fixation of a bone flap following craniotomy has also been known to affect overall surgical outcome.^3^ Titanium mini-plates have been employed for cranial bone flap fixation with excellent results over the years.^4^ Here we present our local experience over a period of three years involving closure of craniotomies with titanium mini plates and give a brief discussion of alternatives available for rigid fixation.

## METHODS

The study was conducted at the Neurosurgery Department of the Combined Military Hospital, Peshawar, Pakistan from March, 2011 to March, 2014. Patients who underwent a craniotomy for supratentorial access for any pathology including tumor excision and aneurysm clipping followed by a cranial bone flap fixation with titanium miniplates were included in the study. Head trauma patients requiring emergency craniotomies were not included since some of these patients required decompressive procedures where the skull flap was not replaced. Craniotomies for posterior fossa approaches are not performed in our unit for lack of specialized equipment. Postoperatively the patients were followed up for a period of 12 months and the results were objectively assessed with visual inspection and radiological imaging to assess for appearance, physical deformity, subsidence or displacement of the bone flap. Subjective data from the patients was not obtained.

**Fig. 1:**
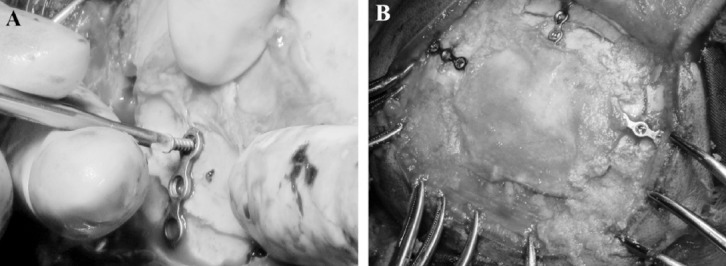
Intraoperative photographs demonstrating the method of fixing the mini titanium plates. It is straightforward and does not consume valuable time in the operating room. Moreover the fixation is completely rigid and not prone to flap settling.

The procedures were carried out by a single neurosurgeon. Craniotomies were done using burr holes which were later filled with bone dust (Fig. 1B). Three or four plates were used to keep the bone flap in place as needed. The authors believe that most cranial bone flaps will not need more than a three point fixation. (Fig. 1A)

## RESULTS

The total number of patients included in the study was 71 with 42 males and 29 females. The age range was two years to 77 years with a mean of 40.07 years and a standard deviation of +/- 18.753 years. All the patients included had supratentorial lesions. Intracranial lesion size ranged from three cm by two cm to seven cm by five cm. Patients requiring infratentorial approaches at our setup required a posterior fossa craniectomy because of unavailability of equipment and hence were not included in the study. The appearance of local incision and contour of the skull were observed on follow up. Patients also underwent a complete neurological exam at each follow up.

**Fig. 2:**
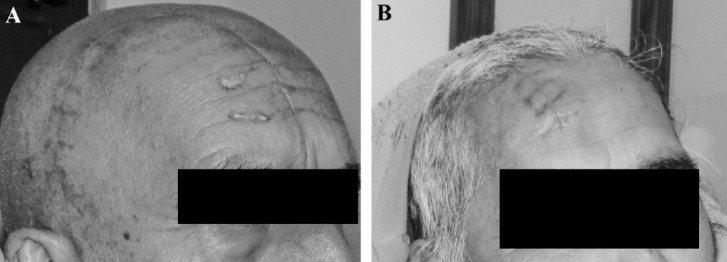
Postoperative photographs taken at follow up demonstrating symmetry of the result. Minor swelling has persisted along the suture line (right). Stitch marks in these case are best avoided by plastic techniques including subcuticular sutures and early removal of any non-absorbable material.

**Fig. 3:**
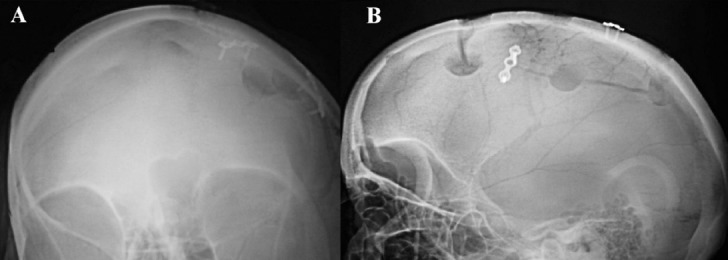
Follow up X ray skull showing even symmetry of the bone flap.

Subcutaneous effusion occurred in two patients who were treated with needle aspiration at day 10. One patient developed a mild postoperative wound infection which was treated with dressings and oral antibiotics. During follow up, skull had good appearance without any discharge and, local deformity or effusion (Fig. 2A and 2B). CT scan and X rays (Fig. 3A and 3B) showed good repositioning of the flap and edge to edge apposition at 2 weeks after operation. Repeat CT/MRI showed no subsidence or displacement of cranial flap or artifacts. However, the metal did cause a maximum of 0.5 cm of image distortion in bot CT and MRI images.

## DISCUSSION

The safety and strength of titanium in the medical industry has been well established. Over the years, it has found extensive use in implants and instruments. Titanium mini-plates were introduced for rigid fixation of cranial bone flaps following craniotomies in 1991 and are currently the most widely used method.^5^ They are currently available in 0.3 mm thickness which does not require prior indentation of the bone to minimize surface relief. This has resulted in excellent functional as well as cosmetic outcomes and faster operating times, reducing the tediousness and uncertainty associated with polypropylene sutures.^1^ The use of sutures was never a safe method of ensuring attachment because it was not sufficiently firm. Cranial flap settling was a very common problem leading to aesthetic deformity. This has been compared with mini titanium paltes and steel wires. A biomechanical evaluation of the three methods demonstrated that sutures were only a third as good in maximal load bearing as the other two methods and hence must be designated to history. 6

Physical disfigurement that is a source of patient distress and cosmetic damage and is usually attribute to either temporalis muscle asymmetry, bone flap depression or a combination of both. These are all more likely to occur following non-rigid fixation of the skull flap.^7^-^9^ Physical disfigurement is not the only complication that should preclude a non-rigid closure because neurological deficits such as constructional apraxia have been known to develop in patients.^10^ Titanium mini-plates have been shown to be superior to stainless steel wires for fixation with a reduced operating time by 40% and less mobility on digital pressure with none of the patients having suboptimal results.^11^ During closure the space left between the skull and the bone flap can be filled with bone powder mixed with the patient’s blood. Bone cementum can give a near perfect appearance postoperatively but is considerably more expensive to use routinely. The importance of cosmetic outcome can be gleaned by the fact that more and more surgeons are using keyhole approaches for major neurosurgical procedures.^12^,^13^

Based on these observations, *Frati A et al* have described an excellent protocol that minimizes scarring, reduces tissue loss and maintains symmetry following craniotomies that we believe should be learnt by young neurosurgeons.^2^ Another option currently available is bioresorbable plates (Bonamates^®^) which are similar to titanium mini plates although significantly more expensive with no significant difference in fracture healing, bone flap sinking or postoperative complications. Study by *lerch and karl-dieter* compared titanium mini-plates with new rivet-like titanium clamp (CranioFix) and both were shown to be superior to suturing.^14^ Similarly in another prospective study by *Broaddus et al* titanium mini-plates were shown to be superior to stainless steel wire for cranium fixation by providing more accurate and rigid re-approximation of the bone edges and the results obtained were comparable to our study.^11^ They should be preferred when the patient requires follow up radiotherapy to avoid dosing adjustments and problems. They do not cause any artifacts either.^15^

Although safe and effective, possible complications associated with the use of titanium hardware include palpability and visibility, which affect the reconstructive aspect of surgery. Other complications include infection, exposure, pain, irritation of nearby nerves and hardware malfunctions.^16^ Removal of hardware is generally restricted to the aforementioned indications and routine removal in pediatric patients is no long practiced since the newer plates have decidedly low toxicity.^17^ However, repeat surgery for removal of hardware is rarely needed when compared to its widespread use. In cases where pain is a reported complication, it becomes critical to assess the origin of the pain and decide whether the plate itself is the cause of the complication or just an innocent bystander.

A newer technique recently described by *Takahashi et al* involves using a single plate under the temporalis muscle in a hairy area by cutting the anterior site of the bone flap at alternate angles. This has been reported to reduce artifacts and provide excellent cosmetic results and has been aptly named the ‘Interdigitated craniotomy’.^18^ In a recent study the risk of infection in mini titanium plates that merited removal was 0.8% in a trauma series. This would imply that the risk of infection and subsequent morbidity/removal was sufficiently small to allow use in low socioeconomic areas.^19^

## CONCLUSION

Titanium mini plates are an excellent tool for instant rigid fixation of cranial bone flaps following craniotomies with preservation of skull symmetry and maintenance of aesthetics as well avoiding complications like floating skull flaps and sinking of the bone flap. It is also simple and more cost effective when compared to complex external fixation systems and bioresobable plate. Currently it appears that the best way to avoid complications is to place hardware away from nerves in a way that does not compromise strength of fixation.

### Authors Contribution

**MJ, SSB, MUR** conceived, designed and did editing of manuscript.

**MJ, MAS, SSB** did data collection and manuscript writing

**MJ, SSB** takes the responsibility and is accountable for all aspects of the work in ensuring that questions related to the accuracy or integrity of any part of the work are appropriately investigated and resolved.
